# Periinterventional Management of Edoxaban in Major Procedures: Results from the DRESDEN NOAC REGISTRY

**DOI:** 10.1055/s-0043-1774304

**Published:** 2023-09-22

**Authors:** Christina Köhler, Luise Tittl, Ulrike Hänsel, Evelyn Hammermüller, Sandra Marten, Christiane Naue, Marianne Spindler, Laura Stannek, Kristina Fache, Jan Beyer-Westendorf

**Affiliations:** 1 Division of “Thrombosis and Hemostasis,” Department of Medicine I, University Hospital “Carl Gustav Carus,” Technical University Dresden, Dresden, Germany

**Keywords:** anticoagulation, edoxaban, bridging, heparin, major procedures

## Abstract

**Background** Edoxaban is a non-vitamin K dependent oral anticoagulant (NOAC) licensed for venous thromboembolism (VTE) treatment or stroke prevention in atrial fibrillation. Major surgical procedures are not uncommon in anticoagulated patients but data on perioperative edoxaban management are scarce.

**Patients and Methods** Using data from the prospective DRESDEN NOAC REGISTRY, we extracted data on major surgical procedures in edoxaban patients. Periinterventional edoxaban management patterns and rates of outcome events were evaluated until day 30 after procedure.

**Results** Between 2011 and 2021, 3,448 procedures were identified in edoxaban patients, including 287 (8.3%) major procedures. A scheduled interruption of edoxaban was observed in 284/287 major procedures (99%) with a total median edoxaban interruption time of 11.0 days (25–75th percentile: 5.0–18.0 days). Heparin bridging was documented in 183 procedures (46 prophylactic dosages, 111 intermediate and 26 therapeutic dosages). Overall, 7 (2.4%; 95% CI: 1.2–4.9%) major cardiovascular events (5 VTE, 2 arterial thromboembolic events) and 38 major bleedings (13.2%; 95% CI: 9.8–17.7%) were observed and 6 patients died (2.1%; 95% CI: 1.0–4.5%). Rates of major cardiovascular events with or without heparin bridging were comparable (4/137; 2.9%; 95% CI: 1.1–7.3% vs. 3/82; 3.7%; 95% CI: 1.3–10.2%). Major bleedings occurred numerically more frequent in patients receiving heparin bridging (23/137; 16.8%; 95% CI: 11.5–23.9%) versus procedures without heparin bridging (9/82; 11.0%; 95% CI: 5.9–19.6%).

**Conclusion** Within the limitations of our study design, real-world periprocedural edoxaban management seems effective and safe. Use of heparin bridging seems to have limited effects on reducing vascular events but may increase bleeding risk.

## Introduction

Edoxaban is a direct-acting non-vitamin K-dependent oral anticoagulant (NOAC) approved for stroke prevention in nonvalvular atrial fibrillation (SPAF) and for the treatment of deep venous thrombosis (DVT) and pulmonary embolism (PE) as well as secondary prevention of venous thromboembolism (VTE). In these indications, NOACs such as edoxaban feature the standard anticoagulation strategy nowadays, widely replacing the former standard vitamin K antagonists (VKAs).

A common clinical problem is the need to perform interventional procedures or surgery in anticoagulated patients, with up to 25% of the patients requiring diagnostic or therapeutic procedures within 2 years. 1 2 For these procedures, uninterrupted continuation of anticoagulation is often not possible for bleeding risk, but interrupting anticoagulation may increase the periprocedural risk of thromboembolic complications. A significant problem with VKA therapy was the slow washout and slow onset of action if VKAs were interrupted for major surgical procedures. The resulting gaps in oral anticoagulation were 2 to 3 weeks, 3 4 5 making interim heparin bridging mandatory in most cases. In contrast, NOACs such as edoxaban exhibit a much shorter half-life (10–14 hours) and a rapid onset of action after restart (maximum plasma levels within 2 hours after intake). 6 This pharmacokinetic profile potentially could help to reduce the periprocedural duration of anticoagulation gaps, allowing for a preprocedural interruption of only 24 to 72 hours and a restart within hours or days after the procedure, as recommended in current guidelines. 7 As a consequence, periprocedural heparin bridging may not be as important in NOAC as in VKA patients. 8 Mounting evidence on this issue has led to several guidelines and expert consensus statements especially in the field of periprocedural anticoagulation of SPAF patients. 7 9 However, data on management patterns and clinical outcomes following major surgical procedures in edoxaban patients are scarce, since edoxaban was the latest NOAC to be approved. Recently published data from the Edoxaban Management in Diagnostic and Therapeutic Procedures (EMIT-AF/VTE) studies 10 11 provide some insights into this topic but less than 25% of the evaluated procedures were high-risk procedures according to the European Heart Rhythm Association (EHRA) classification 7 and complication rates in this subset of procedures were considerably higher than in the minor and low-risk categories.

The objective of this analysis was to evaluate the baseline risk profiles for thromboembolic and bleeding complications, management patterns, and clinical outcomes for edoxaban-anticoagulated patients needing surgical or interventional therapies. With this in mind, we extracted data on major surgical procedures in edoxaban patients treated for SPAF or VTE in the prospective *DRESDEN NOAC REGISTRY.*

## Methods

### Patients

The *DRESDEN NOAC REGISTRY* (Clinical trials.gov: NCT01588119) is a large national prospective observation study in the administrative district of Dresden (Saxony), Germany. An active recruiting network of more than 230 registered physicians and hospitals from the out- and inpatients sector enroll NOAC patients since 2011. Patients on all available NOAC drugs were eligible to participate in the registry, starting from the day of each specific license in Germany. For edoxaban, enrolment started with the license in 2015. All enrolled patients give their written informed consent and are followed by the central registry office with standardized protocols in a prospective manner. Adult patients with SPAF and/or VTE and a planned anticoagulation duration of at least 3 months in a therapeutic dose scheme of edoxaban are eligible. No exclusion criteria apply. Patients are interviewed by telephone visits 30 days after baseline and in a quarterly sequence thereafter to collect data of outcomes and management of NOAC therapy in daily care.

The registry protocol prespecified that all patients undergoing diagnostic or therapeutic procedures were to be interviewed about the periprocedural management of anticoagulation and supporting documents (reports, laboratory values, charts, discharge letters, death certificates) were collected from the health care provider and submitted to central adjudication by the registry office.

### Surgical or Interventional Procedures

All interventional and surgical procedures were categorized according to the bleeding risk categories published in the updated “2021 European Heart Rhythm Association Practical Guide on the Use of Non-Vitamin K Antagonist Oral Anticoagulants” 7 and in the chapter “Perioperative Management of Antithrombotic Therapy” of the 9th American College of Chest Physicians consensus paper, 12 which consider periprocedural bleeding risk, frequency of bleeding, and severity of tissue trauma. The present analyses focuses on major procedures only, defined as procedures with relevant tissue trauma and high bleeding risk and including open pelvic, abdominal and thoracic surgery; brain surgery and neuroinvasive procedures, i.e., spinal tap; major orthopaedic and trauma surgery; extensive wound revision surgery and necrosectomy and vascular surgery.

In case that patients underwent more than one major procedure within the same edoxaban interruption or bridging period, only the first procedure was counted for the 30-day follow-up period and all outcomes during this period were assigned to this index procedure.

### Data Collection and Outcome Evaluation

Data collection included procedure type and date, date and time of last edoxaban intake, type and intensity of heparin bridging anticoagulation, and date and time of restarting edoxaban or any other oral anticoagulant. To qualify for the analysis, edoxaban intake had to be at least once within the last 7 days prior to the index procedure. Low-molecular-weight heparin bridging scheme was classified as prophylactic dose (<100 IU per kg bodyweight per day), intermediate dosage (100–150 IU per kg bodyweight per day), and therapeutic dose regimen (>150 IU per kg bodyweight per day). Unfractionated heparin (UFH) was considered prophylactic if it was administered subcutaneously for maximum of 15,000 IU per day, whereas intravenous UFH infusion with a target activated partial thromboplastin time (aPTT) <2-fold of normal was classified as intermediate dosage and with a target aPTT >2-fold of normal was classified as therapeutic dosage. To evaluate the impact of heparin bridging on clinical outcomes, the use of heparin was categorized into “no heparin bridging (patients with no or low-dose heparin prophylaxis only) versus “heparin bridging” (patients with intermediate or therapeutic dosages of heparin).

Clinical outcomes of interest consisted of major cardiovascular events, major bleeding, and all-cause mortality, respectively.

*Major cardiovascular events* include acute coronary syndrome (comprising unstable angina, non-ST-elevation infarction, and ST-elevation infarction) as well as stroke or transient ischemic attack and systemic embolism complemented by VTE (DVT or PE).
*Major bleedings* were evaluated using the International Society of Thrombosis and Haemostasis (ISTH) definition for major bleeding events 13 and for minor and clinically relevant nonmajor bleedings (CRNMs) with a standardized bleeding assessment tool. 14 Additionally, evaluation of major bleeds was performed using the “Bleeding Academic Research Consortium Definition for Bleeding” (BARC). 15 Of note, the use of the ISTH definition of major bleedings for surgical patients 16 was deemed inappropriate since this definition is to be used in randomized controlled trials to assess efficacy of reversal agents. Supplementary Table S1 (online only) provides an overview over the bleeding assessment and comparisons with published results of the *DRESDEN NOAC REGISTRY* . 17
The *primary outcomes* were a composite endpoint of fatal or nonfatal major cardiovascular events for efficacy and the major bleeding rate for safety evaluation.
*Secondary effectiveness and safety outcomes* were death from cardiovascular disease as well as rates of CRNM bleeding or death from any cause, respectively.

Rates of outcome events were evaluated until day 30 after procedure and data collection included information on outcome severity level, medical approach to periprocedural bleeding complications and to major cardiovascular events.

Statistical analyses were performed for all procedures, including several interventions in the same patient, as well as for subtypes of major procedures.

### Statistics

Demographic and outcome data are shown as absolute values, percentages, standard deviation, and 95% confidence intervals (CIs) or median with 25th and 75th percentiles, when appropriate. 95% CIs for proportions are given according to Clopper–Pearson interval. A *p* -value of ≤0.05 is regarded to be statistically significant. Differences in baseline variables or outcome event rates were compared using the Student's *t* -test, Mann–Whitney U-test, Fisher's exact test, or Chi-squared test, as appropriate.

All statistical analyses were performed using the IBM SPSS Statistics Version 28 and MedCalc version 14.8.1.

## Results

## Baseline Characteristics

Between November 1, 2011 and December 31, 2021, 5,197 patients receiving a NOAC for SPAF or VTE treatment were enrolled. In total, 13,638 surgical or interventional procedures in 3,583 patients were reported in the registry population. Of these, 3,448 procedures were performed in patients who took edoxaban within the preceding 7 days, including 287 (8.3%) major procedures in 245 patients and 3,161 (91.7%) nonmajor procedures in 1,124 patients ( Supplementary Fig. S1 [online only]). Overall, patient characteristics were comparable for major and nonmajor procedures, but significant differences existed with regard to gender, concomitant antiplatelet therapies, and the proportion of patients with an increased risk for stroke or systemic embolism, defined by a CHA _2_ DS _2_ -VASc score ≥2 ( Table 1 ).

**Table 1:** Patient characteristics at baseline of patients with edoxaban undergoing 3,448 surgical or interventional procedures

	All procedures, *N* = 3448	Major procedures, *N* = 287	Nonmajor procedures, *N* = 3,161	*p* -Value
Male, *n* (%)	2,057/3,448 (59.7)	144/287 (50.2)	1,913/3,161 (60.5)	0.0006
Median age (25–75th percentile), y	74.0 (67.0–79.0)	74.0 (67.0–80.0)	74.0 (67.0–79.0)	0.3989
Median BMI (25–75th percentile), kg/m ^2^	28.1 (25.4–31.0)	28.4 (25.4–31.5)	28.1 (25.4–31.0)	0.4425
Indication for edoxaban				
SPAF, *n* (%)	2,816/3,448 (81.7)	237/287 (82.6)	2,579/3,161 (81.6)	0.3764
VTE, *n* (%)	611/3,448 (17.7)	50/287 (17.4)	561/3,161 (17.7)	
Off-label, *n* (%)	21/3,448 (0.6)	0	21/3,161 (0.7)	
Concomitant antiplatelet therapy, *n* (%)	169/3,448 (4.9)	6/287 (2.1)	163/3,161 (5.2)	0.0212
Heart failure, *n* (%)	778/3,448 (22.6)	69/287 (24.0)	709/3,161 (22.4)	0.5316
Arterial hypertension, *n* (%)	2,864/3,448 (83.1)	234/287 (81.5)	2,630/3,161 (83.2)	0.4706
Diabetes, *n* (%)	1,012/3,448 (29.4)	81/287 (28.2)	931/3,161 (29.5)	0.6614
Prior TIA, stroke, or systemic embolism, *n* (%)	342/3,448 (9.9)	24/287 (8.4)	318/3,161 (10.1)	0.3569
PAD/CAD, *n* (%)	567/3,448 (16.4)	52/287 (18.1)	515/3,161 (16.3)	0.4242
Impaired renal function a , *n* (%)	484/3,448 (14.0)	47/287 (16.4)	437/3,161 (13.8)	0.2334
CHADS _2_ ≥ 2 b, *n* (%)	2,126/3,448 (61.7)	179/287 (62.4)	1,947/3,161 (61.6)	0.7960
CHA _2_ DS _2_ -VASc ≥ 2 b , *n* (%)	3,015/3,448 (87.4)	262/287 (91.3)	2,753/3,161 (87.1)	0.0400
CHA _2_ DS _2_ -VASc ≥ 4 b , *n* (%)	1,508/3,448 (43.7)	138/287 (48.1)	1,370/3,161 (43.3)	0.1209
HAS-BLED score ≥ 2 c , *n* (%)	1,824/3,448 (52.9)	152/287 (53.0)	1,672/3,161 (52.9)	0.9826

Abbreviations: BMI, body mass index; PAD/CAD, peripheral arterial occlusive disease/coronary artery disease; SPAF, stroke prevention in atrial fibrillation; TIA, transient ischemic attack; VTE, venous thromboembolism.

a Impaired renal function was defined as current or history of GFR <50 mL/min.

b CHADS _2_ and CHA _2_ DS _2_ -VASc scores are validated risk prediction scores for stroke/systemic embolism in atrial fibrillation patients.

c HASBLED is a validated risk prediction score for major bleeding in anticoagulated patients.

**Fig. 1:**
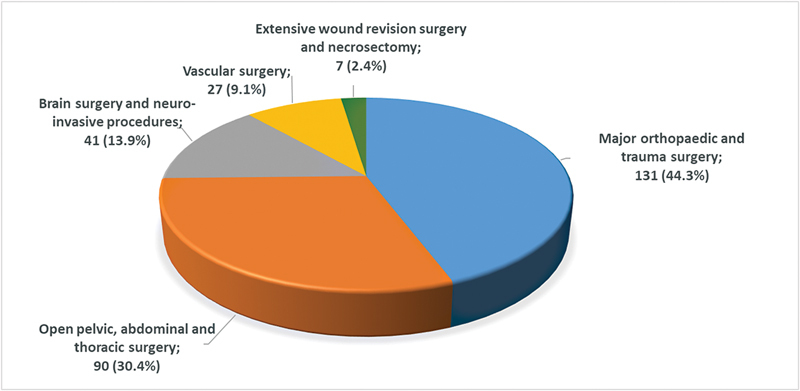
Types of major procedures.

Major procedures consisted of orthopaedic/trauma surgery (44.3%); open pelvic, abdominal or thoracic surgery (30.4%); central nervous system surgery and procedures (13.9%); vascular surgery (9.1%); and extensive wound revision surgery (2.4%) ( Fig. 1 ). Of all 287 procedures, only 3 (1%) were emergency procedures which did not allow for a scheduled preprocedural edoxaban interruption ((intraabdominal abscess; acute cholecystitis; amputation for acute limb ischemia).

In the subset of patients undergoing major procedures, SPAF (81.7%) was the most common indication for edoxaban treatment, followed by VTE therapy and secondary prevention (17.7%) and off-label indications (0.6%). Preprocedure edoxaban dosage was 60 mg once daily in 195 procedures and 30 mg once daily in 92 procedures.

### Patterns of Periprocedural Edoxaban and Bridging Management

Most of the major procedures were performed with a scheduled interruption of edoxaban (284/287; 99%) with a median preprocedural interruption of 2 days (25–75th percentile: 2.0–3.3 days) and a median postprocedural interruption of 8.0 days (25–75th percentile: 2.8–5.0 days). This resulted in a total median edoxaban interruption time of 11.0 days (25–75th percentile: 5.0–18.0 days).

Patients with an eGFR <50 mL/min at baseline were managed with a comparable median preprocedural interruption of 2 days (25–75th percentile: 1.0–3.0 days), but a longer median postprocedural interruption of 13.5 days (25–75th percentile: 6.5–20.3 days).

In a total of 183/287 procedures (63.8%), heparin bridging was used to replace edoxaban anticoagulation, with heparin dosages being prophylactic in 46/183 (25.1%); intermediate in 111/183 (60.6%), or therapeutic in 26/183 (14.2%) of cases ( Supplementary Fig. S1 [online only]). Another 36 (12.5%) procedures were performed without heparin bridging. In the remaining 65 cases, no data on the use and/or dosage of heparin bridging could be obtained from the health care provider.

Supplementary Table S2 (online only) demonstrates baseline characteristics of patients receiving heparin bridging (i.e., intermediate or therapeutic heparin) or not (i.e., no anticoagulation or low-dose heparin prophylaxis only). Both groups had comparable demographic profiles but numerically more patients in the subgroup without heparin bridging had hypertension and a history of stroke, resulting in a higher risk for thromboembolism (as indicated by CHADS _2_ and CHA _2_ DS _2_ -VASc scores ≥2) and bleeding (as indicated by a HAS-BLED score ≥2).

Postprocedure, 237/271 (87.5%) patients with a temporarily edoxaban interruption restarted edoxaban within the 30-day follow-up interval. Thirteen patients permanently discontinued edoxaban, of whom 6/13 (46.2%) were switched to VKA, 4/13 (30.8%) remained on heparin anticoagulation beyond day 30, and 3/13 (23.1%) stopped taking anticoagulants completely.

### Effectiveness and Safety Endpoints

The effectiveness and safety outcomes within 30 days postprocedure are listed in Table 2 . Overall, 7 (2.4%; 95% CI: 1.2–4.9%) major cardiovascular events were reported, comprising 5 newly diagnosed venous thromboembolic events and 2 arterial thromboembolic events (details in Supplementary Table S3 [online only]). In total, 63 bleeding events were observed in 287 major procedures (22.0%; 95% CI: 17.6–2.71%), comprising 38 ISTH major bleeding events (13.2%; 95% CI: 9.8–17.7%, details in Supplementary Table S4 [online only]) and 25 ISTH CRNM bleedings (8.7%; 95% CI: 6.0–12.5%). When criteria within the ISTH major bleeding definition were assessed hierarchically, 1 case was adjudicated as fatal bleeding, 4 cases had critical site bleeding, and 16 had transfusion of ≥2 red blood cell units. The remaining 17 cases had a postprocedural drop in hemoglobin ≥2 g/dL without fulfilling any of the other three criteria. Supplementary Fig. S2 (online only) depicts the distribution pattern over time as well as the BARC bleeding severity within the 38 ISTH major bleeding events.

**Table 2:** Effectiveness and safety outcomes of 287 major procedures in edoxaban patients within day 30 post procedure

Outcome at day 30 postprocedure	Major procedures, *n* = 287
Major CV events, *n* (%; 95% CI)	7 (2.4; 1.2–4.9)
ISTH major bleeding, *n* (%; 95% CI)	38 (13.2; 9.8–17.7)
ISTH nonmajor bleeding, *n* (%; 95% CI)	25 (8.7; 6.0–12.5)
BARC 1, *n* (%; 95% CI)	12 (4.2; 2.4–7.2)
BARC 2, *n* (%; 95% CI)	14 (4.9; 2.9–8.0)
BARC 3a, *n* (%; 95% CI)	22 (7.7; 5.1–11.3)
BARC 3b, *n* (%; 95% CI)	11 (3.8; 2.2–6.7)
BARC 3c, *n* (%; 95% CI)	2 (0.7; 0.2–2.5)
BARC 4, *n* (%; 95% CI)	1 (0.3; 0.1–1.9)
BARC 5a, *n* (%; 95% CI)	0
BARC 5b, *n* (%; 95% CI)	1 (0.3; 0.1–1.9)
All-cause death, *n* (%; 95% CI)	6 (2.1; 1.0–4.5)

Abbreviations: BARC, Bleeding Academic Research Consortium; CI, confidence interval; CV, cardiovascular.

Within 30 days of follow-up, six patients died (2.1%; 95% CI: 1.0–4.5%) with causes of death being a ruptured truncus coeliacus following palliative angioplasty for an infiltrating pancreas cancer (ruled as fatal bleeding), septic organ failure, pneumocystis jirovecii pneumonia, COVID-19-pneumonia, septic complications following clipping of a ruptured cerebrovascular aneurism, or terminal malignant disease. No fatal cardiovascular event occurred.

**Fig. 2:**
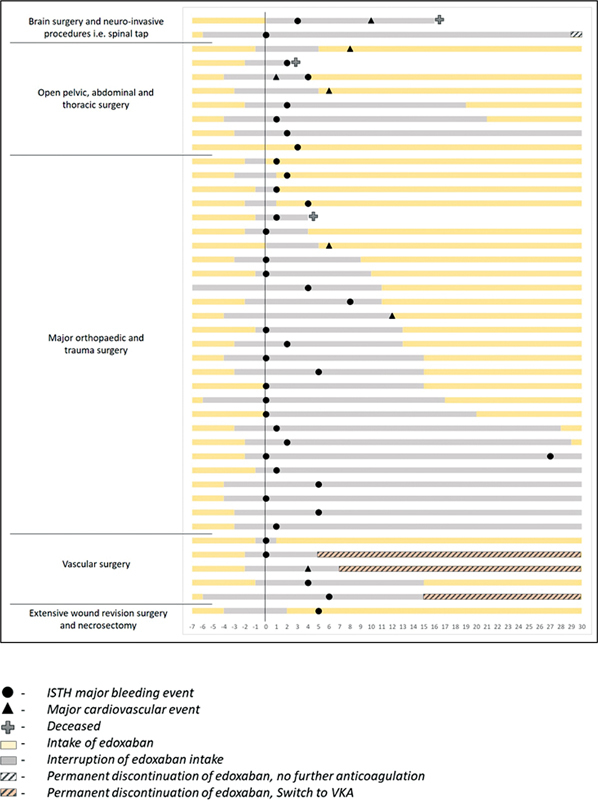
Time–frequency plot of major cardiovascular outcomes, ISTH major bleeding and death in relation to periprocedural edoxaban management, and type of surgery. Of note, the figure depicts only patients who developed clinical outcomes of interest. ISTH, International Society of Thrombosis and Haemostasis.

**Fig. 3:**
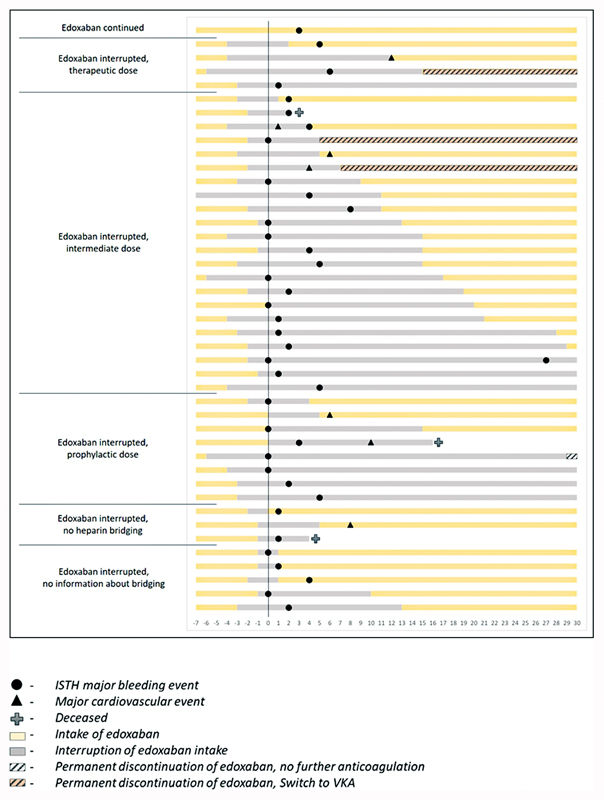
Time–frequency plot of major cardiovascular outcomes, ISTH major bleeding and death in relation to periprocedural edoxaban management, and heparin bridging. Of note, the figure depicts only patients who developed clinical outcomes of interest. ISTH, International Society of Thrombosis and Haemostasis.

The distribution patterns of major cardiovascular outcomes, ISTH major bleeding, and death in relation to periprocedural edoxaban management are depicted in Fig. 2 (according to type of surgical procedure) and Fig. 3 (according to heparin bridging).

As depicted in Figs. 2 and 3 and in Supplementary Table S3 (online only), all seven major cardiovascular events occurred within 2 weeks after procedure, and in 6/7 cases the event was diagnosed during edoxaban interruption or within 24 hours after restarting edoxaban. The remaining major cardiovascular event (partial thrombus in jugular vein at central venous catheter insertion site) was diagnosed on day 8 postsurgery in a patient who restarted edoxaban 30 mg once daily 4 days after hemicolectomy.

The majority of ISTH major bleeding events (20/38; 52.6%) occurred within the first 24 hours after procedure and 37/38 occurred within 8 days (of note, one patient experienced a recurrent major bleeding event on day 27, following an initial major bleeding on the day of surgery). None of the major bleeding events occurred after a restart of edoxaban ( Supplementary Table S4 [online only]).

The use or intensity of heparin bridging did not seem to reduce the risk of major cardiovascular events, which occurred in 4/137 (2.9%; 95% CI: 1.1–7.3%) with heparin bridging versus 3/82 (3.7%; 95% CI: 1.3–10.2%) without ( Table 3 ).

**Table 3:** Effectiveness and safety outcomes of 287 major procedures in edoxaban patients within day 30 postprocedure, according to heparin bridging

Outcome at day 30 after procedure	Edoxaban continued,	Edoxaban interrupted, no heparin bridging (prophylactic low-dose heparin allowed),	Edoxaban interrupted, heparin bridging (intermediate or therapeutic dosages),	Edoxaban interrupted, no information about bridging available,
	*n* = 3	*n* = 82	*n* = 137	*n* = 65
Net clinical benefit (major cardiovascular and major bleeding events), *n* (%; 95% CI)	1(33.3; 6.1–79.2)	12 (14.6; 8.6–23.9)	27 (19.7; 13.9–27.2)	5 (7.7; 3.3–16.8)
Major CV events, *n* (%; 95% CI)	0	3 (3.7; 1.3–10.2)	4 (2.9; 1.1–7.3)	0
Major bleeding, *n* (%; 95% CI)	1 (33.3; 6.1–79.2)	9 (11.0; 5.9–19.6)	23 (16.8; 11.5–23.9)	5 (7.7; 3.3–16.8)
Nonmajor bleeding, *n* (%; 95% CI)	0	4 (4.9; 1.9–11.9)	12 (8.8; 5.1–14.7)	9 (13.8; 7.5–24.3)
BARC 1, *n* (%; 95% CI)	0	2 (2.4; 0.7–8.5)	6 (4.4; 2.0–9.2)	4 (6.2; 2.4–14.8)
BARC 2, *n* (%; 95% CI)	0	1 (1.2; 0.2–6.7)	8 (5.8; 3.0–11.1)	5 (7.7; 3.3–16.8)
BARC 3a, *n* (%; 95% CI)	0	5 (6.1; 2.6–13.5)	13 (9.5; 5.6–15.6)	4 (6.2; 2.4–14.8)
BARC 3b, *n* (%; 95% CI)	1 (33.3; 6.1–79.2)	3 (3.7; 12.5–10.2)	6 (4.4; 2.0–9.2)	1 (1.5; 0.3–8.2)
BARC 3c, *n* (%; 95% CI)	0	2 (2.4; 0.7–8.5)	0	0
BARC 4, *n* (%; 95% CI)	0	0	1 (0.7; 0.1–4.0)	0
BARC 5a, *n* (%; 95% CI)	0	0	0	0
BARC 5b, *n* (%; 95% CI)	0	0	1 (0.7; 0.1–4.0)	0
Death, *n* (%; 95% CI)	0	4 (4.9; 1.9–11.9)	2 (1.5; 0.4–5.2)	0

Abbreviations: BARC, Bleeding Academic Research Consortium; CI, confidence interval; CV, cardiovascular.

With regard to major bleeding, safety outcomes trended to be more frequent in patients undergoing procedures with heparin bridging (23/137; 16.8%; 95% CI: 11.5–23.9%) versus procedures without heparin bridging (9/82; 11.0%; 95% CI: 5.9–19.6%; Table 3 ).

With regard to net clinical benefit (rates of major cardiovascular or major bleeding events combined), Table 3 indicates that event rates were lowest in the subgroup of patients receiving no heparin bridging.

Supplementary Table S5 (online only) demonstrates baseline characteristics of patient with or without major outcome events. Patients experiencing major outcome events tended to be older (median: 77.0 vs. 74.0 years), were more often SPAF patients (88.4 vs. 81.6%), and more often suffered from impaired renal function (23.3 vs. 15.2%), which translated in a higher proportion of patients at higher risk for thromboembolism (as indicated by CHADS _2_ and CHA _2_ DS _2_ -VASc scores ≥2) and bleeding (as indicated by a HAS-BLED score ≥2) at baseline.

As indicated by Supplementary Table S6 (online only), rates of major cardiovascular and bleeding events were considerably higher in patients needing emergency procedures (major cardiovascular events: 9.3%; ISTH major bleeding: 23.3%, and BARC class 3–5 major bleeding: 20.9%) compared with patients after elective major surgery (1.2, 11.5, and 11.1%, respectively).

## Discussion

The present analysis indicates that approximately 8% of all surgical or interventional procedures performed in NOAC patients belong to the high-risk category according to EHRA. 7 This indicates a need for optimal anticoagulant management, since these patients are at increased risk for thromboembolism and bleeding. The thrombotic risk reflects a combination of disposition (hence the indication for anticoagulation) and exposure to major surgery. At the same time, the bleeding risk may be increased by patient-dependent risk factors (such as impaired renal function and frailty), residual anticoagulant activity from NOAC, overlapping anticoagulant activity from heparin bridging, and from the surgical procedure itself. This complex situation indicates a medical need to optimize the periprocedural anticoagulation management of SPAF and VTE patients.

Several studies have therefore addressed the periprocedural management of NOAC patients. One of the largest studies in the field, the “Perioperative Anticoagulation Use for Surgery Evaluation” (PAUSE) trial, 18 reported management patterns and outcomes in more than 3,000 SPAF patients but did not recruit edoxaban patients. In contrast, EMIT-AF/VTE was designed to document the risks of bleeding and thromboembolic events in more than 1,000 patients on edoxaban undergoing diagnostic and therapeutic procedures in clinical practice, but more than 75% of the studied interventions carried a minor or low risk for complications only. 10

Our study therefore provides valuable new insights into the management patterns and outcomes of edoxaban patients undergoing major risk procedures.

### Patient Characteristics

When we compared the patient characteristics of edoxaban patients undergoing major surgical procedures to the profiles of patients undergoing nonmajor procedures, we found that most characteristics were comparable with minor, but significant differences only with regard to female sex, higher CHA _2_ DS _2_ -VASc scores (both more common in major procedures), and concomitant antiplatelet therapy (less common in major procedures). However, advanced age (median 74 years) and high proportion of patients with prior stroke (8–10%), impaired renal function (15%), heart failure (22–24%), or CHA _2_ DS _2_ -VASc score ≥4 (48%) were frequently observed in both subgroups, indicating the high baseline risk of the overall population, which needs to be taken into account when the outcome event rates are discussed below. Overall, the baseline characteristics of our cohort are very similar to those reported in PAUSE (mean age 72–73 years; mean CHA _2_ DS _2_ -VASc score 3.5; prior stroke 7–10%; heart failure 13–19%) 18 and EMIT-AF/VTE (mean age 72 years; CHA _2_ DS _2_ -VASc score >3 41%; renal impairment 19%; heart failure 13%). 10

### Edoxaban Management Patterns

Guidance documents provide specific periprocedural management recommendations for each NOAC and for each risk category of procedures. The EHRA 7 recommendation for edoxaban and major procedures suggests to stop edoxaban 2 days prior without heparin bridging and a restart within 48 to 72 hours postprocedure with a consideration of heparin in prophylactic dosages in between. A similar protocol was prespecified in the PAUSE trial. 18 However, this recommendation is dedicated to SPAF patients and no similarly detailed recommendations are available for VTE patients, who may have a higher thromboembolic risk especially if procedures requiring edoxaban interruption are performed early after VTE diagnosis.

The preprocedural edoxaban interruption in our dataset (median: 2 days, 25–75th percentile: 2.0–3.3 days) indicates a good adherence to the recommendations above. However, given that 15.0% of the major procedures were unplanned events done on the same day of admission and due to the heterogeneity and complexity of some of the observed major procedures, the postprocedural time to the restart of edoxaban showed a wider range with a median of 8 days (25–75th percentile: 2.8–15.0 days), which was even longer for patients with a baseline eGFR <50 mL/min (13.5 days; 25–75th percentile: 6.5–20.3 days).

Colonna et al 10 and Unverdorben et al 11 recently reported on the periprocedural management of edoxaban patients in the EMIT registries. Here, 123 of 280 cases (44%) undergoing high bleeding risk procedures had a preprocedural interruption <48 hours and 30 (11%) had an interruption longer than 72 hours. Unfortunately, no information on the median duration of interruption before procedure or on the time until restart was provided, which limits comparisons with our dataset.

When we compare the management patters for edoxaban with our previously reported mixed NOAC cohort published in the early phase of NOAC use, 17 the management patterns are consistent. In this previous cohort of apixaban, dabigatran, and rivaroxaban patients, the NOAC was stopped 2 days before procedure (interquartile range: 2 days). However, data on the postprocedural NOAC resumption were lacking for the subset of patients undergoing major procedures in this past analysis.

### Efficacy and Safety Outcomes

In our cohort of 287 patients undergoing major surgical procedures, we observed 30-day event rates of 2.4% (major cardiovascular events), 13.2% (ISTH major bleeding), and 2.1% (all-cause mortality), respectively. These numbers clearly reflect the aforementioned fact that our cohort underwent major surgical procedures despite a high-risk profile of baseline cardiovascular and bleeding risk factors. Overall, our outcome data are comparable to those of a previous analysis from our group from the early NOAC era which evaluated 863 procedures in a rivaroxaban cohort (87 major procedures) and reported event rates of 4.6, 8.0, and 2.3% for major cardiovascular events, ISTH major bleeding, and death, respectively. 17

In PAUSE, 18 event rates are more difficult to compare, since three different NOACs were tested in this trial, nearly 70% of procedures were nonmajor procedures and a strict protocol for preprocedural interruption and postprocedural resumption was provided with patients being excluded from the per-protocol analysis in case of protocol deviations. Still, also PAUSE reported a rate of 3% major bleedings following major procedures in DOAC recipients. Unfortunately, cardiovascular events and mortality were not reported separately for patients undergoing major procedures. 18

The observational EMIT registries 10 11 followed SPAF and VTE patients with edoxaban in routine clinical care in a multinational setting and evaluated efficacy and safety in more than 1,000 patients undergoing a wide range of different procedures, including 280 major procedures. Similar to PAUSE, the EMIT registries showed higher rates of bleeding events in major compared with minor procedures (5.7 vs. 3.1%), although only five major events bleeding were documented in total (0.4% of all 1,155 procedures). Rates of arterial events (0.5%) and cardiovascular deaths (0.2%) were also low. Unfortunately, neither major bleeding rates nor VTE event rates can be directly compared with our dataset, since most procedures in EMIT were nonmajor and event rates were not separately provided for the major procedures. However, rates of arterial thromboembolism (1.4%), cardiovascular mortality (0.4%), and all-cause mortality (0.7%) were separately reported for major procedures. Within our composite endpoint of major cardiovascular events, we found two events of arterial thromboembolism (0.7%) and zero cases of cardiovascular deaths, indicating consistency to EMIT. However, our all-cause mortality rate (2.1%) was higher than in the EMIT registries, which was likely unrelated to the edoxaban management and more related to the type of procedures and patient risk profiles, since only one fatal bleeding and zero cardiovascular deaths were contained in this signal.

Finally, when we compare the present outcome rates for edoxaban with our mixed NOAC cohort event rates published in 2014, 17 the crude event rates in the current analysis trended toward lower cardiovascular events (major cardiovascular events 4.6% in 2014 vs. 2.4%; cardiovascular death 2.3% in 2014 vs. 0) but higher rates of major bleeding (8.0% in 2014 vs. 13.2%), although all 95% CIs showed a large overlap.

The 13% rate of major bleeding deserves some further discussion. First of all, the majority of all major bleeding events occurred within the first 48 hours after major procedures and were very likely related to the extent of the surgical procedure itself rather than the periprocedural management of anticoagulants. This consideration is further supported by the fact that 33 of the 38 ISTH major bleeding events were adjudicated based on a need for red blood cell transfusions or a drop in hemoglobin, which are common scenarios also in major procedures in nonanticoagulated patients. This observation further questions if the ITSH bleeding definition is the optimal outcome parameter in this clinical setting. Because of this, all observed bleeding events were additionally adjudicated according to the BARC bleeding definition. Not surprisingly, out of 38 ISTH major bleeding events, only 4 events fell into BARC categories 3c–5, resulting in a crude event rate of 1.4% for immediately life-threatening bleeding. The remaining events fell into categories BARC 3a ( *n* = 22; any transfusion or drop in hemoglobin of 3–5 g/dL) or BARC 3b (drop in hemoglobin >5 g/dL or cardiac tamponade or bleeding requiring surgery or vasopressor drugs).

Finally, we observed a lack of benefit from an intensified heparin bridging regimen. Rates of cardiovascular events were not reduced, but bleeding complications were numerically increased – a finding that is in line with previous observations. 8 17 According to our data, the best risk-benefit relation was observed in patients receiving no heparin bridging or only prophylactic dosages of heparin, which confirms the above mentioned EHRA guidance 7 to use not more than prophylactic heparin dosages if NOAC can be resumed within days after major surgery.

## Limitations

There are several limitations of our study. A selection bias is not negligible, because edoxaban was the last NOAC approved and, as a consequence, the prescription pattern may not be identical to those of other NOAC cohorts from the past. As with all observational studies, missing data or underreporting of outcome events may also be confounders in our registry. However, our methodology has been well standardized over more than 10 years; all patient interviews during follow-up are performed by well-trained study nurses using standardized questionnaires. For the reported 287 procedures, no patient was lost to follow-up (0%) or withdrew consent (0%). Although not part of our outcome endpoint for the presented analysis, nonmajor bleeding events are routinely collected in our registry, thus limiting the likelihood that major events were missed.

Furthermore, with only 45 major outcome events (7 major cardiovascular events, 38 major bleeding events) our study did not allow for adjusted comparisons of different management strategies. It is in the nature of observational studies that the potential for confounding prevents establishing causal relationships between prescribed treatments and outcomes. For this, randomized comparisons for different treatment strategies are needed. The value of observational studies in this field is to provide insights into management patterns and to study outcomes within these patterns. As such, we provide very granular data on the periprocedural edoxaban management, the intensity of heparin treatments, and provide patterns for the timing of outcome events according to type of procedures or heparin strategies. On the other hand, our sample size of >3,000 procedures in edoxaban patients including 287 major procedures, the use of clinically relevant endpoints, and the rigorous central event adjudication process are important strengths of our registry and the presented analysis.

## Conclusion

Our results indicate that adherence to bridging recommendations in edoxaban-treated patients undergoing major surgical procedures is adequate. Given the high baseline risk of the population and the increase of thrombotic and bleeding risks from major surgery, the observed complication rates fell into a clinically acceptable range. Edoxaban management patterns (stop 2 days before and resumption within days postsurgery) follow existing guidelines and the best risk–benefit balance was achieved by guideline-recommended low-dose heparin prophylaxis instead of therapeutic heparin bridging. Our results therefore not only confirm previous studies in this field and guidance recommendations in SPAF patients, but extend these to VTE patients and to patients receiving edoxaban—a cohort for which real-world data are still scarce. Still, the observed high degree of variance especially for using heparin bridging and for delaying restart of oral anticoagulation postsurgery warrants further studies, investigating the reasons for these clinical decisions and to evaluate optimized hospital-standard operating procedures that aim to further improve standardization and patient outcomes.
