# Supplementary material for: Periinterventional Management of Edoxaban in Major Procedures: Results from the DRESDEN NOAC REGISTRY
Source: TH Open. 2023 Sep 22;7(3):e251–61. doi: 10.1055/s-0043-1774304 (PMC10516686; doi:10.1055/s-0043-1774304)
Supplement: Supplementary file 1 — Supplementary Material [file 10-1055-s-0043-1774304-s23030010.pdf]

Supplementary Materials

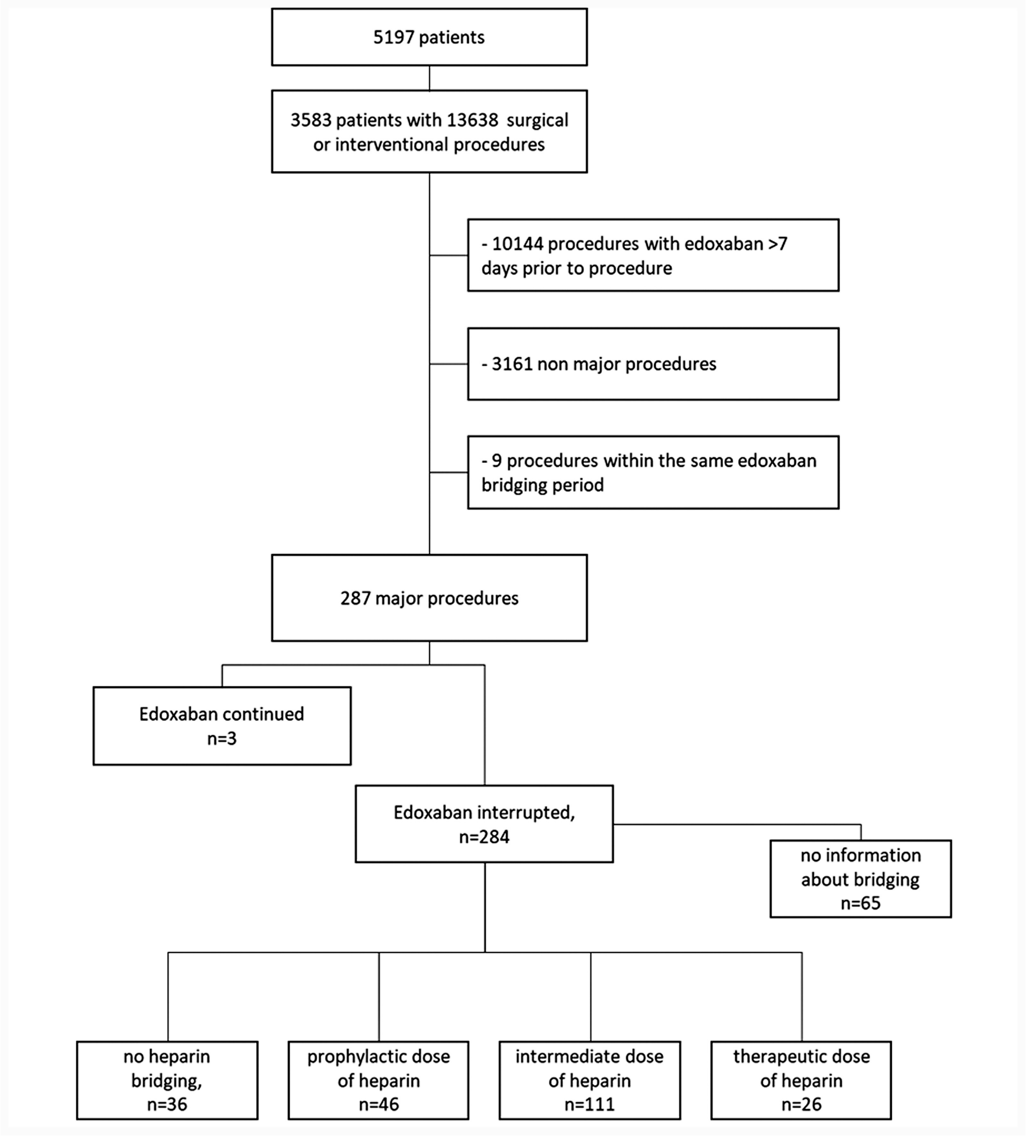

Supplementary Fig. S1 Flowchart of study cohort.

 © 2023. The Author(s).

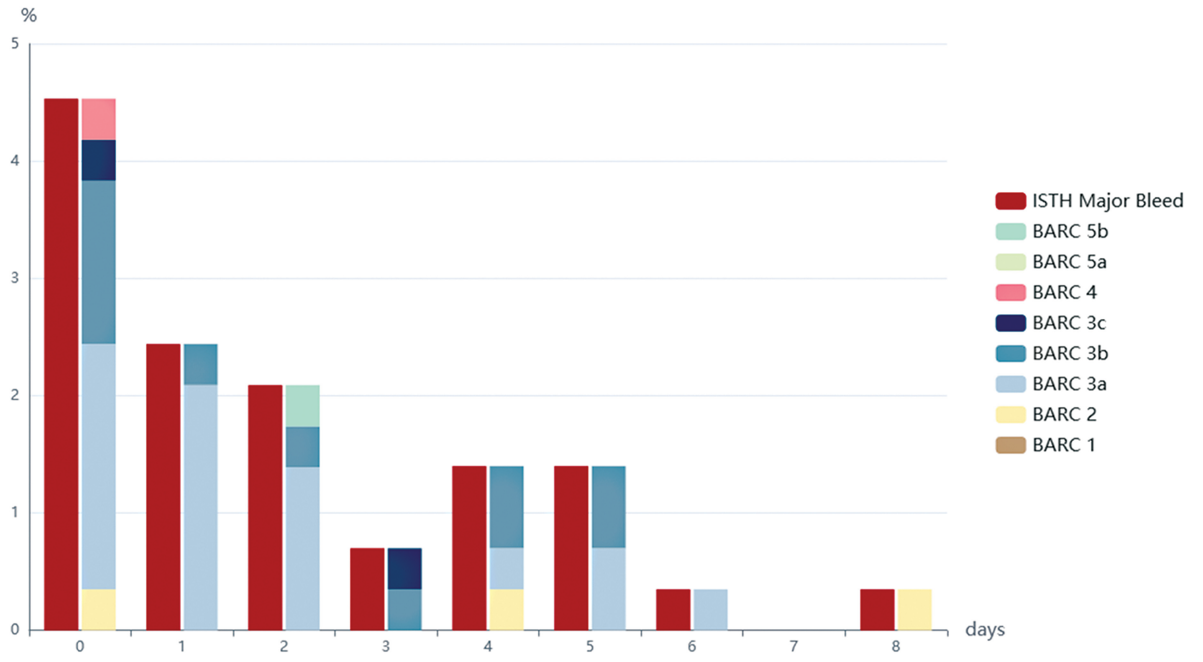

Supplementary Fig. S2 Distribution of ISTH major bleeding over time (all events occurred between days 0–8 postprocedure) and correlation to BARC bleeding severity. BARC, Bleeding Academic Research Consortium; ISTH, International Society of Thrombosis and Haemostasis.

Supplementary Table S1 Bleeding definitions of BARC,[1] ISTH/SSC,[2] and as centrally adjudicated in the DRESDEN NOAC registry

DRESDEN NOAC	ISTH		BARC definition	
No event		No overt bleeding event	Type 0	No bleeding
Nonmajor bleeding	Minor bleeding	All not clinically relevant nonmajor hemorrhages	Type 1	Not actionable; no unscheduled studies, hospitalization, or treatment; self-limiting bleedings
	Clinically relevant minor bleeding	Overt bleeding requiring medical attention, i.e., hospitalization, surgical or medical treatment, or change in antithrombotics	Type 2	Overt actionable bleeding that requires nonsurgical or medical treatment or hospitalization, increased level of care, and rapid evaluation
Major bleeding	Major bleeding	Bleeding + drop in Hb level of $\geq$1.24 mmol/L (2 g/dL) or transfusion $\geq$2 units of whole blood or red cells	Type 3a	Overt bleeding + decrease of Hb level of 1.86–3.10 mmol/L (3–5 g/dL); any overt bleeding + transfusion
		Overt symptomatic bleeding in a critical area or organ such as pericardial, retroperitoneal, intraarticular, intramuscular with compartment syndrome	Type 3b	Overt bleeding + decrease of Hb level of $\geq$3.10 mmol/L ($\geq$5 g/dL); cardiac tamponade, hemorrhage requiring surgical intervention, or intravenous vasoactive agents for bleeding management
		Overt symptomatic bleeding:<br>- Intracranial<br>- Intraspinal<br>- Intraocular	Type 3c	Intraocular bleeding with impaired vision. Intracranial bleeding including intraspinal and subcategories confirmed by autopsy, imaging, or lumbar puncture. Excludes microbleeds or hemorrhagic transformation.
			Type 4	Severe CABG-related bleeding
		Fatal bleeding	Type 5 a	Probable fatal bleeding without autopsy or imaging, but clinically suspicious
			Type 5 b	Definite fatal bleeding with an overt bleeding or confirmed by autopsy or imaging

Abbreviations: BARC, Bleeding Academic Research Consortium; CABG, coronary artery bypass graft; Hb, hemoglobin; ISTH, International Society of Thrombosis and Haemostasis.

 © 2023. The Author(s).

Supplementary Table S2 Patient characteristics at baseline of patients undergoing major surgical procedures with edoxaban interruption and with or without heparin bridging

	All major procedures with edoxaban interruption, *N* = 219	Without heparin bridging, *N* = 82	With heparin bridging, *N* = 137
Male, *n* (%)	115/219 (52.5)	44/82 (53.7)	71/137 (51.8)
Median age (25–75th percentile), y	74.0 (67.0–80.0)	75.0 (68.0–80.0)	74.0 (67.0–79.0)
Median BMI (25–75th percentile), kg/m^2	28.3 (25.3–31.2)	29.1 (26.3–32.4)	27.7 (25.0–30.8)
Indication for edoxaban			
SPAF, *n* (%)	180/219 (82.2)	69/82 (84.1)	111/137 (81.0)
VTE, *n* (%)	39/219 (17.8)	13/82 (15.9)	26/137 (19.0)
Off-label, *n* (%)	0	0	0
Concomitant antiplatelet therapy, *n* (%)	4/219 (1.8)	0	4/137 (2.9)
Heart failure, *n* (%)	57/219 (26.0)	23/82 (28.0)	34/137 (24.8)
Arterial hypertension, *n* (%)	176/219 (80.4)	74/82 (90.2)	102/137 (74.5)
Diabetes, *n* (%)	66/219 (30.1)	25/82 (30.5)	41/137 (29.9)
Prior TIA, stroke, or systemic embolism, *n* (%)	18/219 (8.2)	9/82 (11.0)	9/137 (6.6)
PAD/CAD, *n* (%)	40/219 (18.3)	16/82 (19.5)	24/137 (17.5)
Impaired renal function[a], *n* (%)	38/219 (17.4)	14/82 (17.1)	24/137 (17.5)
$CHADS_2 \geq 2$, *n* (%)	135/219 (61.6)	58/82 (70.7)	77/137 (56.2)
CHA_2DS_2-VASc ≥ 2, *n* (%)	200/219 (91.3)	79/82 (96.3)	121/137 (88.3)
CHA_2DS_2-VASc ≥ 4, *n* (%)	102/219 (46.6)	43/82 (52.4)	59/137 (43.1)
HAS-BLED score ≥ 2, *n* (%)	121/219 (55.3)	51/82 (62.2)	70/137 (51.1)

Abbreviations: BMI, body mass index; PAD/CAD, peripheral arterial occlusive disease/coronary artery disease; SPAF, stroke prevention in atrial fibrillation; TIA, transient ischemic attack; VTE, venous thromboembolism.
[a]Impaired renal function was defined as current or history of GFR <50 mL/min.

Supplementary Table S3 Characteristics and outcomes of patients with major cardiovascular events postprocedure

Gender, age	Indication for edoxaban therapy	Procedure	Edoxaban interruption (days before procedure)	Heparin bridging	Time between procedure and outcome (days)	Outcome
m, 70 y	SPAF	Hemicolectomy	4	Intermediate-dose LMWH	1	PE
m, 74 y	SPAF	Thrombectomy and peripheral bypass surgery	2	Intermediate-dose UFH	4	Early occlusion of peripheral bypass
f, 52 y	VTE	Oncoplastic surgery in breast cancer	3	Intermediate-dose LMWH	6	DVT progression
f, 80 y	SPAF	Repositioning and osteosynthesis of humerus fracture	0	Prophylactic-dose LMWH	6	PE from a pre-existing DVT
f, 81 y	SPAF	Hemicolectomy	1	None	8	Partial thrombosis of jugular vein at CVC insertion site
f, 86 y	SPAF	Intracerebral aneurysm clipping	0	Prophylactic-dose LMWH	10	Ischemic stroke

 © 2023. The Author(s).

Supplementary Table S3 (*Continued*)

Gender, age	Indication for edoxaban therapy	Procedure	Edoxaban interruption (days before procedure)	Heparin bridging	Time between procedure and outcome (days)	Outcome
f, 84 y	SPAF	Reimplantation of a total knee arthroplasty	4	Therapeutic-dose rivaroxaban	12	DVT

Abbreviations: CVC, central venous catheter; DVT, deep vein thrombosis; LMWH, low-molecular-weight heparin; PE, pulmonary embolism; SPAF, stroke prevention in atrial fibrillation; UFH, unfractionated heparin.

Supplementary Table S4 Characteristics and outcomes of patients with ISTH major bleeding events postprocedure

Gender, age	Indication for edoxaban therapy	Procedure	Edoxaban interruption (days before procedure)	Heparin bridging	Time between procedure and outcome (days)	Outcome
m, 74 y	SPAF	Transsphenoidal malignoma biopsy	6	Prophylactic-dose LMWH	0	Local ICH after biopsy
m, 74 y	SPAF	Total hip arthroplasty	6	Intermediate-dose LMWH	0	Postoperative surgical site bleeding
m, 77 y	SPAF	Total hip arthroplasty	1	Indefinite dose of LMWH	0	Postoperative anemia
w, 79 y	SPAF	Total hip arthroplasty	3	Intermediate-dose LMWH	0	Postoperative anemia
w, 81 y	SPAF	Aortic valve replacement with biological prosthesis	1	Indefinite dose of LMWH	0	Intraoperative vascular bleeding
m, 71 y	SPAF	Total hip arthroplasty	4	Intermediate-dose LMWH	0	Intraoperative bleeding
w, 71 y	SPAF	Osteosynthesis of femoral neck fracture	2	Intermediate-dose UFH	0	Postoperative anemia
m, 44 y	VTE	Total hip arthroplasty	2	Prophylactic-dose LMWH	0	Postoperative anemia
m, 83 y	SPAF	Osteosynthesis of femur fracture	0	Intermediate-dose LMWH	0	Perioperative blood loss with postoperative anemia
m, 67 y	SPAF	Aortic valve replacement with biological prosthesis	2	Intermediate-dose LMWH	0	Postoperative surgical site bleeding
w, 80 y	SPAF	Total knee arthroplasty	4	Prophylactic-dose LMWH	0	Postoperative bleeding anemia, local hematoma
w, 87 y	SPAF	Osteosynthesis of femur fracture	0	Prophylactic-dose LMWH	0	Postoperative bleeding
w, 82 y	SPAF	Osteosynthesis of femoral neck fracture	1	Intermediate-dose LMWH	0	Intraoperative bleeding with anemia
m, 68 y	SPAF	Total hip arthroplasty	2	None	1	Perioperative surgical site bleeding and local hematoma

(*Continued*)

 © 2023. The Author(s).

Supplementary Table S4 (*Continued*)

Gender, age	Indication for edoxaban therapy	Procedure	Edoxaban interruption (days before procedure)	Heparin bridging	Time between procedure and outcome (days)	Outcome
m, 67 y	VTE	Hemicolectomy	4	Intermediate-dose LMWH	1	Wound drainage bleeding
w, 77 y	SPAF	Total hip arthroplasty	1	Indefinite dose and duration	1	Postoperative anemia
m, 84 y	SPAF	Osteosynthesis of pertrochanteric femur fracture	1	None	1	Postoperative anemia
w, 84 y	SPAF	Osteosynthesis of tibial fracture	1	Intermediate-dose LMWH	1	Postoperative anemia
m, 76 y	SPAF	Osteosynthesis of spine injury	3	Therapeutic-dose LMWH	1	Postoperative anemia
m, 79 y	SPAF	Total hip arthroplasty	3	Intermediate-dose LMWH	1	Postoperative surgical site bleeding
m, 84 y	SPAF	Cancer surgery of mandibular cancer	2	Intermediate-dose LMWH	2	Postoperative local hematoma
w, 77 y	SPAF	Total hip arthroplasty	2	Intermediate-dose UFH	2	Postoperative surgical site bleeding
m, 79 y	SPAF	Radical cystectomy surgery	3	Prophylactic-dose LMWH	2	Anemia after radical cystectomy
w, 74 y	SPAF	Total hip arthroplasty	3	Intermediate-dose LMWH	2	Intraoperative bleeding
w, 82 y	SPAF	Total knee arthroplasty	3	Indefinite dose of LMWH	2	Postoperative anemia
w, 68 y	SPAF	Laparotomy with pancreatic cancer surgery	2	Intermediate-dose LMWH	2	Hemorrhagic shock due to rupture of celiac truncus
w, 86 y	SPAF	Intracerebral aneurysm clipping (neuro surgical)	0	Prophylactic-dose LMWH	3	ICH after external ventricular drainage
w, 74 y	SPAF	Laparotomy for cholecystitis	No interruption	No interruption	3	Gastrointestinal bleeding
m, 70 y	SPAF	Hemicolectomy for cancer surgery	4	Intermediate-dose LMWH	4	Bleeding of colon anastomosis
w, 68 y	VTE	Total hip arthroplasty	2	Indefinite dose and duration	4	Perioperative bleeding with anemia
m, 74 y	SPAF	Crural bypass surgery	1	Intermediate-dose LMWH	4	Postpuncture bleeding with large hematoma
w, 84 y	SPAF	Revision surgery of total hip arthroplasty	7	Intermediate-dose LMWH	4	Postoperative bleeding anemia
m, 80 y	SPAF	Total knee arthroplasty	3	Prophylactic-dose LMWH	5	Joint bleeding

 © 2023. The Author(s).

Supplementary Table S4 (*Continued*)

Gender, age	Indication for edoxaban therapy	Procedure	Edoxaban interruption (days before procedure)	Heparin bridging	Time between procedure and outcome (days)	Outcome
m, 63 y	SPAF	Ankle joint arthrodesis	3	Intermediate-dose LMWH	5	Postoperative hematoma ankle joint
w, 88 y	SPAF	Cancer surgery with melanoma resection	4	Therapeutic-dose LMWH	5	Postoperative vaginal bleeding
w, 71 y	VTE	Explantation of a septic knee endoprosthesis	4	Intermediate-dose LMWH	5	Intra- and postoperative anemia
m, 78 y	SPAF	Aortic valve replacement with mitral and tricuspid valve reconstruction	6	Therapeutic-dose LMWH	6	Postoperative anemia
w, 72 y	SPAF	Osteosynthesis of a distal femoral fracture	2	Intermediate-dose UFH	8	Postoperative bleeding anemia

Abbreviations: DVT, deep vein thrombosis; ICH, intracranial hemorrhage; LMWH, low-molecular-weight heparin; PE, pulmonary embolism; SPAF, stroke prevention in atrial fibrillation; UFH, unfractionated heparin.

Supplementary Table S5 Patient characteristics at baseline of edoxaban patients with or without major bleeding or cardiovascular events following major surgical procedures

	All major procedures, $N = 287$	Without major outcome, $N = 244$	With major outcome, $N = 53$
Male, *n* (%)	144/287 (50.2)	124/244 (50.8)	20/43 (46.5)
Median age (25–75th percentile), y	74.0 (67.0–80.0)	74.0 (66.0–78.0)	77.0 (71.0–81.5)
Median BMI (25–75th percentile), kg/m^2	28.4 (25.4–31.5)	28.6 (25.7–31.8)	27.3 (25.1–30.9)
Indication for edoxaban			
SPAF, *n* (%)	237/287 (82.6)	199/244 (81.6)	38/43 (88.4)
VTE, *n* (%)	50/287 (17.4)	45/244 (18.4)	5/43 (11.6)
Off-label, *n* (%)	0	0	0
Concomitant antiplatelet therapy, *n* (%)	6/287 (2.1)	6/244 (2.5)	0
Heart failure, *n* (%)	69/287 (24.0)	53/244 (21.7)	16/43 (37.2)
Arterial hypertension, *n* (%)	234/287 (81.5)	201/244 (82.4)	33/43 (76.7)
Diabetes, *n* (%)	81/287 (28.2)	71/244 (29.1)	10/43 (23.3)
Prior TIA, stroke, or systemic embolism, *n* (%)	24/287 (8.4)	19/244 (7.8)	5/43 (11.6)
PAD/CAD, *n* (%)	52/287 (18.1)	44/244 (18.0)	8/43 (18.6)
Impaired renal function[a], *n* (%)	47/287 (16.4)	37/244 (15.2)	10/43 (23.3)
$CHADS_2 \geq 2$, *n* (%)	179/287 (62.4)	150/244 (61.5)	29/43 (67.4)
CHA_2DS_2-VASc ≥ 2, *n* (%)	262/287 (91.3)	222/244 (91.0)	40/43 (93.0)
CHA_2DS_2-VASc ≥ 4, *n* (%)	138/287 (48.1)	115/244 (47.1)	23/43 (53.5)
HAS-BLED score ≥ 2, *n* (%)	152/287 (53.0)	124/244 (50.8)	28/43 (65.1)

Abbreviations: BMI, body mass index; PAD/CAD, peripheral arterial occlusive disease/coronary artery disease; SPAF, stroke prevention in atrial fibrillation; TIA, transient ischemic attack; VTE, venous thromboembolism.
[a]Impaired renal function was defined as current or history of GFR <50 ml/min.

 © 2023. The Author(s).

Supplementary Table S6 Effectiveness and safety outcomes of edoxaban patients within day 30 after elective versus emergency surgical procedures

Outcome at day 30 postprocedure	Major procedures, *n* = 287	Major elective procedures, *n* = 244	Major emergency procedures, *n* = 43
Major CV events, *n* (%; 95% CI)	7 (2.4; 1.2–4.9)	3 (1.2; 0.4–3.6)	4 (9.3; 3.7–21.6)
ISTH major bleeding, *n* (%; 95% CI)	38 (13.2; 9.8–17.7)	28 (11.5; 8.1–16.1)	10 (23.3; 13.2–37.8)
ISTH nonmajor bleeding, *n* (%; 95% CI)	25 (8.7; 6.0–12.5)	25 (10.2; 7.0–14.7)	0
BARC 1, *n* (%; 95% CI)	12 (4.2; 2.4–7.2)	12 (4.9; 2.8–8.4)	0
BARC 2, *n* (%; 95% CI)	14 (4.9; 2.9–8.0)	13 (5.3; 3.1–8.9)	1 (2.3; 0.4–12.1)
BARC 3a, *n* (%; 95% CI)	22 (7.7; 5.1–11.3)	16 (6.6; 4.1–10.4)	6 (14; 6.6–27.3)
BARC 3b, *n* (%; 95% CI)	11 (3.8; 2.2–6.7)	9 (3.7; 2.0–6.9)	2 (4.7; 1.3–15.5)
BARC 3c, *n* (%; 95% CI)	2 (0.7; 0.2–2.5)	1 (0.4; 0.1–2.3)	1 (2.3; 0.4–12.1)
BARC 4, *n* (%; 95% CI)	1 (0.3; 0.1–1.9)	1 (0.4; 0.1–2.3)	0
BARC 5a, *n* (%; 95% CI)	0	0	0
BARC 5b, *n* (%; 95% CI)	1 (0.3; 0.1–1.9)	1 (0.4; 0.1–2.3)	0
All-cause death, *n* (%; 95% CI)	6 (2.1; 1.0–4.5)	2 (0.8; 0.2–2.9)	4 (9.3; 3.7–21.6)

Abbreviations: CI, confidence interval; CV, cardiovascular.

References

1 Mehran R, Rao SV, Bhatt DL, et al. Standardized bleeding definitions for cardiovascular clinical trials: a consensus report from the Bleeding Academic Research Consortium. Circulation 2011;123(23):2736–2747

2 Schulman S, Kearon CSubcommittee on Control of Anticoagulation of the Scientific and Standardization Committee of the International Society on Thrombosis and Haemostasis. Definition of major bleeding in clinical investigations of antihemostatic medicinal products in non-surgical patients. J Thromb Haemost 2005;3(04):692–694

 © 2023. The Author(s).
